# Ankrd45 Is a Novel Ankyrin Repeat Protein Required for Cell Proliferation

**DOI:** 10.3390/genes10060462

**Published:** 2019-06-16

**Authors:** Yunsi Kang, Haibo Xie, Chengtian Zhao

**Affiliations:** 1 Institute of Evolution & Marine Biodiversity, Ocean University of China, Qingdao 266003, China; kangyunsi@stu.ouc.edu.cn (Y.K.); xiehaibo@stu.ouc.edu.cn (H.X.); 2 Laboratory for Marine Biology and Biotechnology, Qingdao National Laboratory for Marine Science and Technology, Qingdao 266003, China; 3 Ministry of Education Key Laboratory of Marine Genetics and Breeding, College of Marine Life Sciences, Ocean University of China, Qingdao 266003, China

**Keywords:** ANKRD45, zebrafish, KRAS, cell cycle, apoptosis, cancer

## Abstract

Ankyrin repeats, the most common protein–protein interaction motifs in nature, are widely present in proteins of both eukaryotic and prokaryotic cells. Ankyrin repeat-containing proteins play diverse biological functions. Here, we identified the gene *ankrd45,* which encodes a novel, two ankyrin repeat-containing protein. Zebrafish *ankrd45* displayed a tissue specific expression pattern during early development, with high expression in ciliated tissues, including otic vesicles, Kupffer’s vesicles, pronephric ducts, and floor plates. Surprisingly, zebrafish *ankrd45* mutants were viable and developed grossly normal cilia. In contrast, mutant larvae developed enlarged livers when induced with liver specific expression of Kras^G12V^, one of the common mutations of KRAS that leads to cancer in humans. Further, histological analysis suggested that multiple cysts developed in the mutant liver due to cell apoptosis. Similarly, knockdown of *ANKRD45* expression with either siRNA or CRISPR/Cas9 methods induced apoptosis in cultured cells, similar to those in zebrafish *ankrd45* mutant livers after induction. Using different cell lines, we show that the distribution of ANKRD45 protein was highly dynamic during mitosis. ANKRD45 is preferably localized to the midbody ring during cytokinesis. Together, our results suggest that ANKRD45 is a novel ankyrin repeat protein with a conserved role during cell proliferation in both zebrafish embryos and mammalian cells.

## 1. Introduction

The ankyrin repeat (ANK), a motif of 33 amino acid residues, is one of the most common protein motifs that is widely present in all forms of life, including bacteria, archaea, eukaryotes, and viruses. ANK proteins often contain such motifs in tandem arrays with each exhibiting a canonical helix-turn-helix conformation, a well-known motif involved in protein–protein interactions [1,2]. ANK repeats were first identified in the yeast cell cycle regulator Swi6/Cdc10 and the *Drosophila* cell signaling Notch protein, and later named after the human membrane-associated ankyrin protein, which contains 24 such repeats and regulates the interaction between the cytoskeleton and the plasma membrane [3,4].

Currently, thousands of ANK-containing proteins have been identified, which perform a wide range of functions, including signal transduction, cell cycle regulation, vesicle trafficking, cytoskeletal organization, and transcriptional regulation [2,5]. Dysfunction of ANK proteins is associated with many human disorders. Mutation of p16, a tumor suppressor protein with four ankyrin repeats, is associated with several human cancers due to abnormal cell cycle defects [6,7]. Disruption in the ankyrin repeat domains in Notch proteins leads to neurological disorders in humans [8,9]. The ANK protein IκB, an inhibitor of nuclear factor kappa B (NF-κB), is involved in transcription regulation and mediates metabolism and inflammatory responses [10,11]. IκB may also induce apoptosis in cancer cells as inhibition of IKKa, an IκB kinase leading to its degradation, can switch the effects of estrogens on human breast cancer MCF-7 cells from anti- to pro-apoptotic [12,13]. Inversin (INVS), also known as NPHP2, is a ciliary-localizing protein with multiple ANK domains. Patients harboring mutations in the *INVS* gene manifest multiple defects, including renal cystic disease and left-right asymmetry defects due to abnormal functioning of cilia [14].

Cilia are tiny organelles protruding from the cell surface and perform diverse biological functions [15]. Dysfunction of cilia may lead to multiple defects during embryonic development and result in a class of genetic disorders collectively termed as ciliopathies [16]. Recently, zebrafish have been used as disease models for ciliopathies [17]. Cilia are present in various organs of developing zebrafish larvae. Particularly, the olfactory pits, pronephric ducts, floor plates, and Kupffer’s vesicles are tissues rich in motile cilia, and cilia genes are often expressed at a higher level in these organs [17]. Zebrafish cilia mutants frequently develop curly body axis phenotype due to motile cilia defects in the spinal cord [18].

KRAS, together with HRAS and NRAS, are members of the RAS family of small GTPases and mutations of these RAS genes are associated with one third of human cancers [19]. The *KRAS^G12V^* mutation is one of the common mutations that is found in many human cancers [19,20]. The G12V oncogenic mutation renders the KRAS protein more active by diminishing its hydrolysis from the GTP-bound active state to the GDP-bound inactive state. The GTP-bound KRAS^G12V^ proteins chronically bind to and activate multiple downstream signaling pathways, including MAPK or PI3K/AKT signals, which lead to excessive cell proliferation and subsequent carcinogenesis [20].

In this study, we report the functions of a novel ANK protein, ANKRD45. We show that *ankrd45* exhibits a tissue specific expression pattern with high enrichment in ciliary tissues during early zebrafish development. Although zebrafish *ankrd45* mutants were viable with grossly normal cilia, mutant larvae displayed proliferation defects when induced with a liver specific *kras^G12^^V^* transgene. We further investigated the role of ANKRD45 both in zebrafish and in cell lines. Our data suggests that ANKRD45 is a novel player during cell cycle regulation.

## 2. Materials and Methods 

### 2.1. Zebrafish Strains

All zebrafish strains were maintained at a 14 h light/10 h dark cycle at 28.5 °C. The Tet-on inducible double transgene *Tg (fabp10:rtTA2s-M2;TRE2:EGFP-kras^G12V^)* (Gift from Dr. Gong, NUS) was used to generate the liver tumor model [21,22]. The *ankrd45* mutants were generated using the CRISPR/Cas9 system with the following target sequencing for sgRNA synthesis: 5′-GGTGTCCAGCTGACCCCACA-3′. 

### 2.2. Whole Mount In Situ Hybridization and Immunohistochemistry

Full-length *ankrd45* gene was amplified from 24-h post fertilization (hpf) zebrafish cDNA with the following primers: Forward 5′-CACACCACATCACTACTCTTC-3′, Reverse 5′-GTAATGCAGTCCAACAGTTTC-3′. The PCR products were ligated into pEASY-T3 vectors. Probe preparation and hybridization were performed using standard protocols. To analyze its expression in liver, zebrafish larvae were anaesthetized and fixed at 5 days post fertilization (dpf) for cryosectioning. Transverse sections through the liver were collected for fluorescence in situ hybridization analysis. Fluorescent signal amplification using a TSA-plus Fluorescein System (Perkin Elmer Life Sciences, Boston, MA, USA) was carried out according to the manufacturer’s protocols. For immunohistochemistry, the anti-monoglycylated Tubulin Antibody (TAP952, Merck-Millipore, Darmstadt, Germany) was used to visualize cilia in wild-type and mutant larvae. The antibody staining procedure was performed as described previously [23].

### 2.3. Pharmaceutical Treatments and mRNA Overexpression

To evaluate liver development, we generated wild-type and *ankrd45* homozygous mutants carrying the *Tg (fabp10:rtTA2s-M2;TRE2:EGFP-kras^G12V^)* transgene. In our experience, we have found that the homozygous EGFP-*kras^G12V^* transgene developed larger livers compared with the heterozygote transgene in wild-type larvae. To exclude this situation, we crossed *ankrd45* homozygous mutants carrying the EGFP-*kras^G12V^* transgene with *ankrd45* homozygous mutants, and compared them with wild-type embryos carrying the heterozygote transgene. The collected embryos were treated with 60 μg/mL doxycycline hydrochloride (Sangon Biotech, Shanghai, China) or DMSO starting from 60 hpf. Images of the liver with green fluorescence were captured using a Leica M165FC microscope at different time points after treatment. The size of liver was evaluated using Image J software according to the area of green fluorescent protein (GFP) expression.

For rescue experiments, full length *ankrd45* cDNA was subcloned into pCS2+ expression vectors. After linearization, mRNA was transcribed using the mMessage mMachine Sp6 transcription kit (Invitrogen-Ambion, Austin, TX, USA). Microinjections were performed at the one-cell stage at a concentration of 100 ng/μL. Injected embryos were maintained for drug treatments at later stages to evaluate liver development.

### 2.4. Histological Analysis and TUNEL Assay

Zebrafish larvae carrying the EGFP-*kras^G12V^* transgene were fixed at three days after doxycycline treatment in 4% paraformaldehyde (PFA) (*w*/*v*) in PBST overnight at 4 °C. After gradually dehydrating in ethanol, the fixed embryos were finally embedded in JB4 embedding medium (Polysciences Inc., Warrington, PA, USA). Transverse sections through the liver were collected using Leica RM2235 microtome and stained with hematoxylin and eosin. Images were taken using a Leica DM2500 microscope. For TUNEL analysis, the doxycycline-treated embryos were fixed in 4% PFA overnight at 4 °C, infiltrated in 30% sucrose overnight at 4 °C, then embedded in frozen medium for cryosectioning using LEICA cryostat (CM1860). The sections were collected using adhesive slides and air dried for one hour at room temperature (RT). After that, sections were incubated in 70% ethanol/30% glacial acetic acid at −20 °C for 5 min, washed 5 times with 50 mM Tris-HCl, then blocked for 30 min at RT with blocking solution (1× PBS/10% normal goat serum/0.5% TrionX-100). The TUNEL assay was performed using the in situ cell death detection kit (Roche, Indianapolis, IN, USA) according to standard protocols from the manufacturer.

### 2.5. BrdU Incorporation Assay

At 68 h after doxycycline inducement, zebrafish larvae were incubated in 10 mM BrdU (Sigma, St. Louis, MO, USA) in 1% DMSO for four hours at 28.5 °C, then anesthetized in tricaine and fixed with 4% paraformaldehyde for two hours at RT. The fixed embryos were then sent for cryosectioning. Sections were treated with 2 N HCl for 1 h at RT, blocked 1 h with blocking solution, then incubated with mouse anti-BrdU antibody (Invitrogen) overnight at 4 °C. The next day, after a 1-h wash with PBST, the sections were further incubated with secondary goat anti-mouse IgG antibody. For cell culture analysis, Hep3B cells were incubated with BrdU at a final concentration of 20 μM for 2 h. After a PBS wash, cells were fixed with 4% PFA for 5 min, permeabilized with acetone:methanol (1:1) for 3 min on ice, then rehydrated in PBS for 5 min. The following denaturing and antibody incubation steps were similar to those of the staining of the cryosections.

### 2.6. Cell Culture

Hep3B, Hek293, U2OS, and HeLa cells were cultured in 90% Dulbecco’s modified Eagle medium (DMEM; Gibco) supplemented with 10% fetal bovine serum (FBS; Gibco, Grand Island, NY, USA), and maintained at 37 °C in a humidified incubator supplied with 5% CO_2_. The hTERT-RPE-1 cells were grown in DMEM/F12 (Gibco) media supplemented with 10% FBS.

### 2.7. RNA Interference and Immunofluorescence

SiRNAs against human *ANKRD45,* were purchased from GenePharma (Shanghai, China). The four siRNA sequences were: siRNA1: 5′-GCCCAAGAACCAGAGGAAA-3′, siRNA2: 5′-GGAUCCUGAGAAUCCUCAU-3′, siRNA3: 5′-GGAAGAAAGGGCUCGAGAU-3′, and siRNA4: 5′-CCUUAAGGAAGACAAGAA-3′. A general sequence of 5′-UUCUCCGAACGUGUCACGU-3′ was used as a negative control. All siRNAs were transfected at a final concentration of 50 μM with Lipofectamine™ RNAiMAX (Invitrogen) following the manufacturer’s protocol. The following antibodies were used for Western blot analysis: rabbit polyclonal antibodies against human ANKRD45 (Abcam, ab97878, Cambridge, MA, USA), cleaved PARP (Cell Signaling, Danvers, MA, USA), and actin (Beyotime Biotech Inc., Beijing, China).

For immunofluorescence, cells were fixed in 4% PFA at RT for 10 min, then treated with cold methanol at −20 ℃ for 5 min. After two washes with PBS, the slides were permeabilized using 0.25% Triton-X-100 for 5 min, blocked with 3% BSA in PBS for 45 min, then incubated with anti-ANKRD45 and anti-α-tubulin antibodies in 3% BSA in PBS overnight at 4 ℃. After washing with PBS, the cells were incubated with secondary antibodies for 4 h at RT. All the images were captured using a Lecia Sp8 confocal microscope. We used two antibodies (Abcam, ab97878 and Invitrogen, PA5-21796) to detect ANKRD45 localization in cultured cells. Both of these two antibodies exhibited a similar staining pattern to that of ANKRD45 proteins. We only showed the immunofluorescence images in the paper with anti-ANKRD45 antibodies from Abcam (Cambridge, MA, USA).

### 2.8. Knockdown of ANKRD45 with the CRISPR/Cas9 System

Two sgRNAs that target the human *ANKRD45* gene were designed with the following sequences: sgRNA-1: 5′-CTTAGAAGAAGACATCGTT-3′ and sgRNA-2: 5′-TTGTAAAAGGGGGTTCTTA-3′. The target sequences were cloned into the sgRNA expression vector via BbsI restriction enzyme digestion. Each gRNA expression construct was transfected together with the pSpCas9 expression vector into Hep3B cells. The empty sgRNA expression vector was transfected together with pSpCas9 as a negative control. After 48 h of transfection, the expression level of ANKRD45 was examined by immunoblot analysis.

### 2.9. Time-Lapse Assay

HeLa cells were first transfected with control or *ANKRD45* siRNA for 48 h as described above. After that, cells were plated on 20 mm glass-bottomed dishes. Time-lapse images were recorded at 5 min intervals for 12 h using a Nikon A1 laser scanning confocal microscope equipped with 20× objective lens. The movies were generated using the Image J software (NIH Image, Bethesda, MD, USA).

### 2.10. CCK8 Assay

Cell proliferation was assessed using the CCK-8 assays kit (Sigma), which uses a water-soluble tetrazolium salt to quantify the number of live cells by producing an orange formazan dye upon bio-reduction in the presence of an electron carrier. Briefly, Hep3B cells were transfected with 50 μM control or *ANKRD45* siRNA and seeded onto 96-well plates at an initial density of 1 × 10^3^ cells/well. At 1, 2, 3, and 4 days, 10 μL of CCK-8 solution was added to each well of the plate. After a one-hour incubation at 37 °C, spectrophotometric readings (A450 nm) were obtained on a microplate reader (Bio-Rad Laboratories, Hercules, CA, USA). Each experiment was repeated at least three times, and the data presented represent the mean of all measurements.

### 2.11. Cell Cycle and Annexin V Apoptosis Assay

Cell cycle distributions were determined by flow cytometry through staining of the cells with propidium iodide (PI). Briefly, Hep3B cells were harvested at 48 h or 72 h after siRNA treatment, then washed with 1× PBS and resuspended in 75% cold ethanol at 4 °C overnight. After a further wash with 1× PBS, fixed cells were treated with RNase for 30 min, and incubated with 30 μg/mL PI for one hour in the dark. The cells were analyzed with a Beckman Coulter Cytomics FC500MPC Flow Cytometry Analyzer.

The percentage of apoptotic cells was assessed with an eBioscience™ Annexin V-FITC Apoptosis Detection Kit according to the manufacturer’s instructions (Invitrogen, 88-8005-72). Hep3B cells transfected with siRNA or sgRNA constructs were harvested and washed with cold PBS, then resuspended in 100 μL binding buffer containing 5 μL Annexin V-FITC. After 15 min incubation in the dark, cells were centrifuged at 1000 rpm for 5 min and resuspended in 500 μL binding buffer containing 5 µL of PI for 15 min in the dark at RT. The apoptosis was analyzed by a Beckman Coulter Cytomics FC500MPC Flow Cytometry Analyzer.

## 3. Results

### 3.1. Expression of Ankrd45 Is Enriched in Ciliated Organs

**Figure 1:**
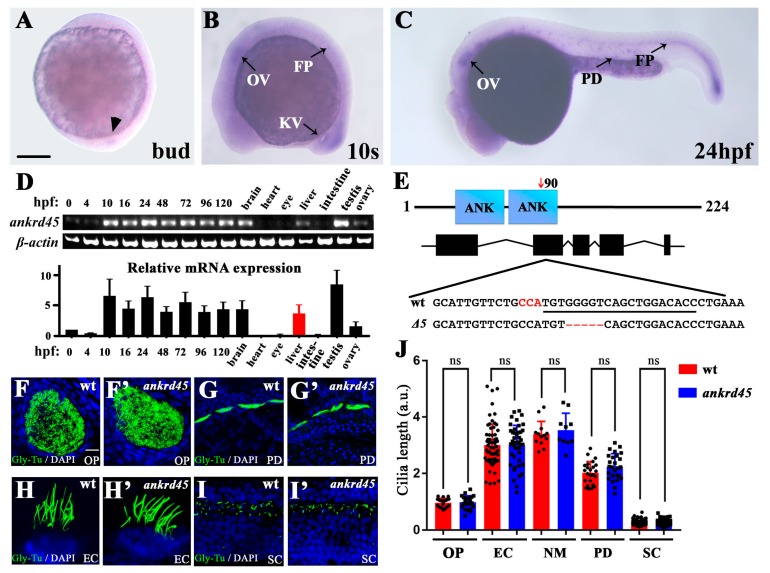
Expression pattern of *ankrd45* and cilia development in *ankrd45* mutants. (**A**–**C**) Whole-mount in situ hybridization results showing the expression of *ankrd45* at different stages. (**A**) At the bud stage, *ankrd45* is expressed in the dorsal forerunner cells (arrowhead). (**B**) At the 10-somite stage, *ankrd45* is expressed in the otic vesicle (OV), Kupffer’s vesicle (KV), and floor plate (FP). (**C**) *ankrd45* is enriched in the otic vesicle, the pronephric duct (PD), and the floor plate at 24 hpf. (**D**) Real-time PCR showing the expression of *ankrd45* at different stages or adult tissues as indicated. Bottom: bar graph showing the relative expression levels of *ankrd45* at different stages or adult tissues as indicated. (**E**) Diagram showing the genomic structure of the zebrafish *ankrd45* gene. Bottom shows the sequences of wild-type and mutant alleles (Δ5). Underlined sequence indicates the spCas9 target region, and the PAM sequence is indicated in red. (**F**–**I’**) Confocal images showing the cilia in the olfactory pit (**F**,**F’**), pronephric duct (**G**,**G’**), ear cristae (**H**,**H’**), and spinal cord (**I**,**I’**) in wild-type or mutant larvae visualized with anti-glycylated tubulin antibody. (**J**) Bar graph showing the length of cilia per arbitrary unit (a.u.) in different organs as indicated. OP, olfactory pit; EC, ear cristae; SC, spinal cord; NM, neuromast. Scale bars: 200 μm in A–C; 20 μm in F–I’. ns, not significant.

When searching for genes involved in ciliogenesis, we identified *ankrd45* as a candidate gene using whole-mount in situ-hybridization gene expression screening. The staining of *ankrd45* was first weakly detected in the dorsal forerunner cells at 80% epiboly to bud stages (Figure 1A). At the 10-somite stage, strong staining was detected in the otic placodes, Kupffer’s vesicles, and floor plates (Figure 1B). Later, at 24 h post-fertilization (hpf), *ankrd45* was mainly expressed in those organs rich in motile cilia, including otic vesicles, pronephric ducts, and floor plates (Figure 1C) [17]. At later stages, *ankrd45* displayed a ubiquitous expression pattern. Real-time PCR results showed that the expression of *ankrd45* persists into the adult stage with higher expression in the brain, liver, and testis (Figure 1D).

### 3.2. Ankrd45 Is Dispensable for Zebrafish Development

Zebrafish Ankrd45 contains 224 amino acids with two ankyrin repeats, which are conserved between humans and zebrafish (Figure 1E). The expression pattern suggests that Ankrd45 might play a role during ciliogenesis. To elucidate this, we generated zebrafish *ankrd45* mutants using the CRISPR/Cas9 system and recovered a mutant allele containing a 5 bp deletion in the target area (Figure 1E). This deletion introduced a new restriction site in the target region, and led to a premature termination of translation that encodes only the N-terminal 90 amino acids of the wild-type protein (Figure S1A,B). Further in situ hybridization results suggested that mutant mRNA was degraded due to nonsense-mediated mRNA decay, a common phenomenon in zebrafish mutants (Figure S1C,D) [24,25].

Next, we compared cilia in several organs of mutant and wild-type larvae, including olfactory pits, pronephric ducts, spinal canals, otic vesicles, and neuromasts, by immunofluorescence with anti-glycylated tubulin antibodies. Surprisingly, cilia developed normally in these tissues, and the length of mutant cilia was comparable to those in wild type (Figure 1F,J). Furthermore, *ankrd45* mutants were viable and fertile. These results suggest that Ankrd45 is dispensable for ciliogenesis, as well as zebrafish development.

### 3.3. Liver Specific Expression of Kras^G12V^ Induced Larger Livers in Ankrd45 Mutants

**Figure 2:**
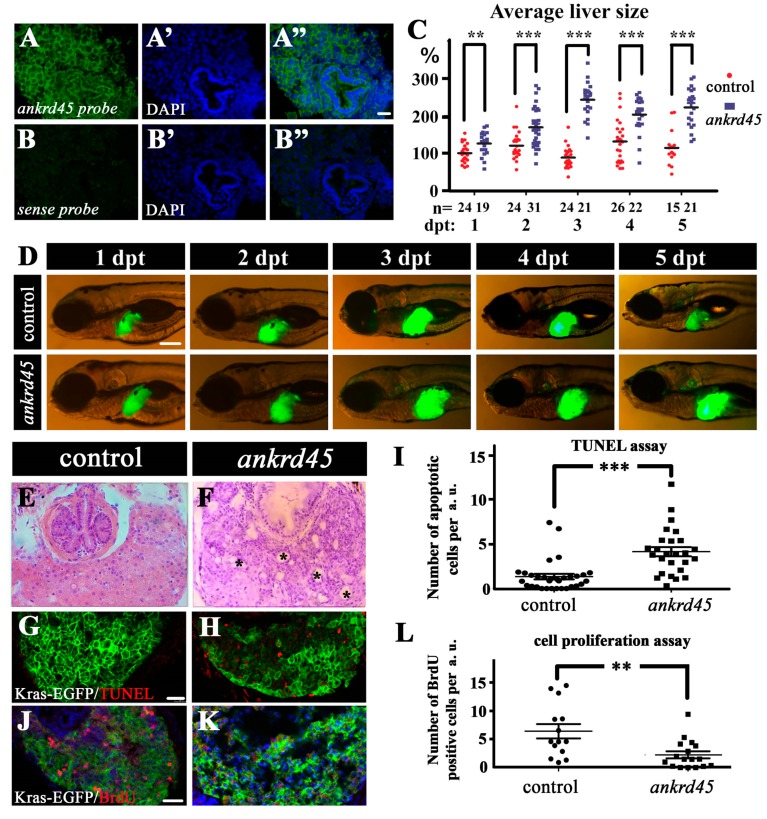
Liver-specific induction of *Kras^G12V^* results in larger livers in *ankrd45* mutants. (**A**–**B”**) Fluorescent in situ hybridization results showing the expression of *ankrd45* in the liver of 5 dpf wild-type zebrafish larvae. Sense probe of *ankrd45* was used as a negative control (**B**–**B”**). (**C**) Statistical results showing the average size of livers in control larvae (*EGFP-kras^G12V^* heterozygote transgene) or *ankrd45* larvae (*ankrd45* homozygous mutants carrying *EGFP-kras^G12V^* heterozygote transgene) at different time points after doxycycline treatment as indicated. Each dot represents the size of one larva. The average size of the livers at 1 day post treatment (dpt) in the control larvae was set as 100%. The number of larvae examined is shown at the bottom. (**D**) Representative images showing EGFP-Kras^G12V^ expression in the livers of control and *ankrd45* mutants at different time points after doxycycline treatment. (**E**,**F**) Hematoxylin and eosin staining results showing the multiple cyst-like regions (asterisks) in the livers of *ankrd45* mutants after treatment. (**G**,**H**) Confocal images showing the apoptotic cells stained by the TUNEL assay. (**I**) Dotted graph showing the number of apoptotic cells in the livers of control and *ankrd45* mutants 3 days after treatment. (**J**,**K**) Confocal images showing BrdU positive cells in the livers of control and mutant larvae as indicated. (**L**) Dotted graph showing the number of BrdU positive cells in the livers of control and *ankrd45* mutants 3 days after treatment. Scale bars: 25 μm in panels A–B”, 200 μm in panel D, and 25 μm in panels G–K. ** *p* < 0.01, *** *p* < 0.001.

Considering that many ANK proteins play a role during cell cycle regulation, we investigated whether loss of function of Ankrd45 could lead to cell proliferation defects in zebrafish larvae. Both real-time PCR and fluorescence in situ hybridization results suggested that *ankrd45* displayed a higher expression level in the liver (Figure 1D, Figure 2A–B”). We further generated *ankrd45* mutants carrying a doxycycline-inducible transgenic line, Tg (*fabp10:rtTA2s-M2*; *TRE2:EGFP-kras^G12V^*), which contained a liver-specific double transgene to induce the expression of EGFP-Kras^G12V^ specifically in the liver [21,22]. KRAS is one of the three RAS isoforms that is most frequently mutated in human cancers. The KRAS^G12V^ mutation reduces the ability of the RAS small GTPase to hydrolyze to GDP, rendering it constitutively active for downstream signaling [22,26]. Upon induction, as reported previously, enlarged and hyperplastic livers developed in both wild-type and *ankrd45* mutant larvae (Figure 2C,D). We compared the size of the liver between mutant and wild-type larvae from day 1 to day 5 after doxycycline treatment. Unexpectedly, *ankrd45* mutants developed a larger liver than wild-type larvae after treatment (Figure 2C,D). The average liver size was almost double in *ankrd45* mutants compared to that in control larvae (Figure 2C). We further injected wild-type *ankrd45* mRNA into the mutant larvae and found that the formation of the enlarged liver was diminished in the mutants (Figure S2A). These data suggested that Ankrd45 protein is involved in the Kras^G12V^-induced liver tumor growth.

### 3.4. Enlarged Cystic Liver Is Due to Cell Degeneration in Kras^G12V^-Induced Ankrd45 Mutant

To better understand the mechanisms underlying the enlarged liver development in the mutant, we further investigated the liver morphology using hematoxylin and eosin (H&E) staining and oil red O staining on the histological sections. The oil red O staining showed no difference in lipid formation between mutant and wild-type embryos (data not shown). In wild-type embryos, H&E staining results showed a hyperplastic liver due to Kras^G12V^–induced cell proliferation. In contrast, multiple cyst-like areas were present throughout the mutant liver (Figure 2E,F). To further explain the reason for cyst formation, we compared cell apoptosis between mutant and wild-type larvae at different time points after induction. TUNEL assay results showed that the number of apoptotic cells increased significantly (*p* < 0.001) in the mutant liver at 3, 4, and 5 days post treatment (dpt) (Figure 2G–I, Figure S2B,D). In contrast, BrdU labeling results showed that the number of proliferating cells decreased substantially in the mutant (Figure 2J–L, Figure S2C,E). Noticeably, we observed significant loss of proliferating cells at five-days post treatment, compared with those at 3 and 4 dpt (Figure S2E). These data suggest that loss of *ankrd45* results in a large number of apoptotic cells at the expense of proliferating cells in the Kras^G12V^-induced liver tumor, which leads to the formation of large areas of cystic degeneration and represents the main reason for enlarged livers in the mutants.

### 3.5. Dynamic Subcellular Localization of ANKRD45 During Mitosis

**Figure 3:**
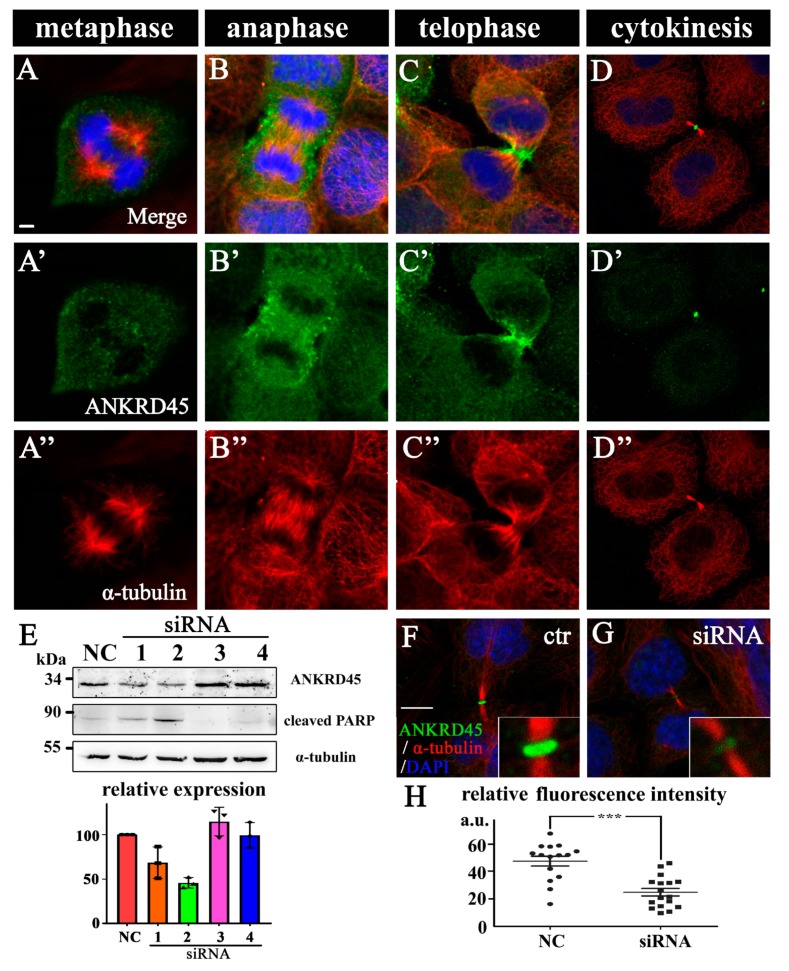
Subcellular localization of ANKRD45 during mitosis. (**A**–**D”**) Subcellular localization of ANKRD45 during mitosis in human hepatoma Hep3B cells. The distribution of ANKRD45 was visualized with anti-ANKRD45 antibody (green). The cytoskeleton was stained by anti-α-tubulin antibody (red) and nuclei were labeled with DAPI (blue). (**E**) Hep3B cells were treated with control or four siRNAs against *ANKRD45* and analyzed with Western blotting using ANKRD45 antibody or a cleaved form of the PARP antibody. The α-tubulin antibody was used as a loading control. Bottom bar graph shows the statistical results of ANKRD45 expression in control or siRNA-treated cells from the immunoblots results. (**F**,**G**) Confocal images showing the localization of ANKRD45 in the midbody during cytokinesis in control (**F**) or siRNA#2 (**G**) treated HeLa cells. (**H**) Quantitative analysis of relative ANKRD45 expression in the midbody ring of control or siRNA#2-treated HeLa cells. NC, negative control. Scale bars: 5 μm. *** *p* < 0.001.

Considering that high levels of Kras^G12V^ expression can lead to hepatocellular carcinoma (HCC) in zebrafish and that KRAS^G12V^ is also the most popular mutation driving cancer cell proliferation [19,22], we further analyzed the role of ANKRD45 using several human cancer cells. First, we analyzed subcellular localization of ANKRD45 using human hepatoma Hep3B cells. Interestingly, the subcellular localization of ANKRD45 was cell-cycle dependent. Although we did not detect any signals during interphase, ANKRD45 localized diffusely in the entire cytoplasm during metaphase (Figure 3A–A”). During anaphase and telophase, ANKRD45 was detected to be highly concentrated at the cleavage furrow region (Figure 3B–C”). ANKRD45 appeared to accumulate rapidly during cleavage-furrow formation and was enriched at the midbody ring at the later cytokinesis (Figure 3D,D”). Of note, ANKRD45 was distributed in a dotted manner at early stages of cell division and was later enriched in the apical region beneath the cell membrane, similar to those proteins that are essential for midbody ring formation [27,28]. We observed a similar localization pattern of ANKRD45 in several other cell lines, suggesting the conserved role of this protein during cell division (Figure S3A–D). Furthermore, we pre-incubated the antibody with recombinant human ANKRD45 peptide before antibody staining, such treatment completely blocked the fluorescence signal in the midbody (Figure S3E,F).

### 3.6. Loss of ANKRD45 Results in Cell Proliferation Defects in Cultured Cells

To further examine the role of ANKRD45 during cell division, we designed four *ANKRD45* siRNAs to silence *ANKRD45*’s expression. siRNA#2 showed more than 50% knockdown efficiency compared to other siRNAs in both Hep3B cells and HeLa cells (Figure 3E, Figure S3G,H). When treated with this siRNA, the fluorescence signal of ANKRD45 in the midbody ring decreased significantly (*p* < 0.001) (Figure 3F–H), which further confirmed the localization of ANKRD45.

**Figure 4:**
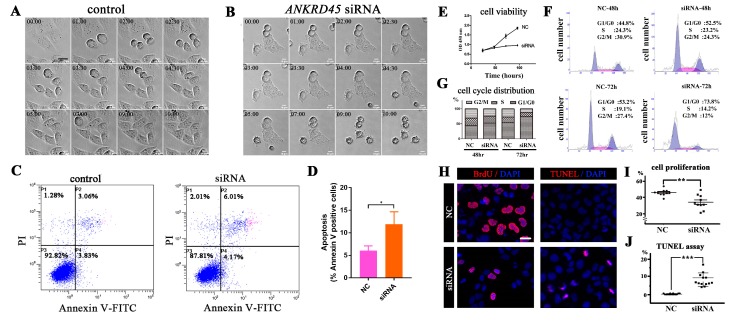
Knockdown of ANKRD45 results in cell cycle defects**.** (**A**,**B**) DIC (Differential Interference Contrast) still images obtained from time-lapse microscopy showing HeLa cells undergoing cell division. Compared with control siRNA-treated cells (**A**), knockdown of ANKRD45 (**B**) resulted in the delay of cell division and cell death. (**C**,**D**) Flow cytometry analysis of Annexin V-FITC and propidium iodide staining of the control and *ANKRD45* siRNA-treated Hep3B cells. The percentage of apoptotic cells increased significantly after siRNA treatment (**D**). (**E**) Cell proliferation of Hep3B cells analyzed by CCK-8 assay. (**F**,**G**) Flow cytometry cell cycle analysis of Hep3B cells treated with control or siRNA. The percentage of cells in different phases of the cell cycle are shown in (G). (**H**) Confocal images showing proliferating and apoptotic Hep3B cells visualized by BrdU incorporation and the TUNEL assay. (**I**) Quantitative results of the percentage of BrdU positive cells in control or siRNA-treated Hep3B cells. (**J**) Quantitative results of the percentage of apoptotic cells from the TUNEL assay. NC, negative control. Scale bars: 25 μm. * *p* < 0.05, ** *p* < 0.01, *** *p* < 0.001.

Surprisingly, we observed robust, round-shaped, detached cells floating in the medium in siRNA-treated cells, indicating that these were apoptotic cells. To better describe this observation, we performed time-lapse microscopy to compare cell division between control and *ANKRD45* siRNA-treated HeLa cells. In control siRNA-treated cells, cell cycle progressed through mitosis and cytokinesis normally within 2–3 h (Figure 4A, Supplementary Movie S1). In contrast, silencing of *ANKRD45* resulted in cell division defects and cell death characterized by rounding up of the cell body and eventual detachment from the plate (Figure 4B, Supplementary Movie S2). Consistent with this, the cleaved form of PARP-1, a hallmark of cell apoptosis, was also increased in both HeLa and Hep3B cells (Figure 3E, Figure S3G). We stained these cells with Annexin V and performed flow cytometry analysis, which further confirmed cell apoptosis after siRNA treatment (Figure 4C,D).

Next, we performed a cell proliferation assay using a cell counting kit-8 (CCK-8) to evaluate cell viability in *ANKRD45* siRNA-treated cells. Compared with the control group, knockdown of *ANKRD45* expression dramatically affected cell viability (Figure 4E). Flow cytometry analysis results suggested that the percentages of cells in G0/G1 stages were greatly increased at both 48 h and 72 h after treatment, suggesting that knockdown of *ANKRD45* expression delayed cell cycle progression (Figure 4F,G). Finally, BrdU incorporation and TUNEL assay further suggested that cell proliferation was diminished and cell apoptosis was induced after siRNA treatment (Figure 4H–J).

**Figure 5:**
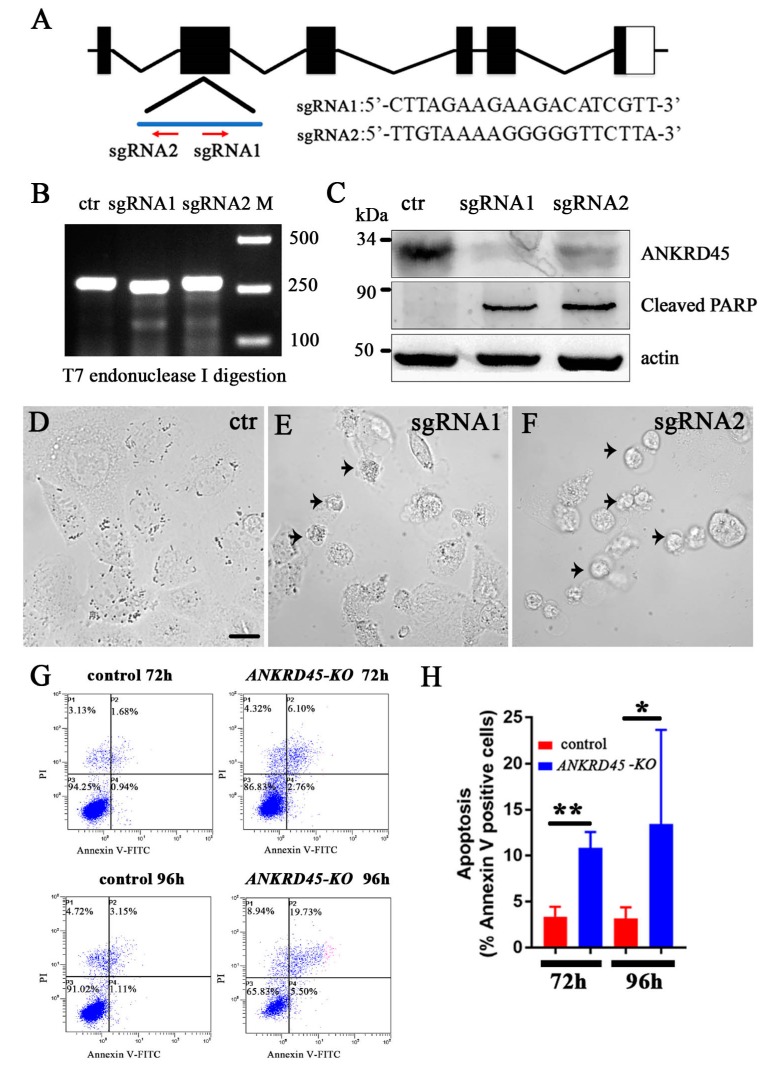
Transient knockdown of ANKRD45 with the CRISPR/Cas9 system results in cell apoptosis. (**A**) Genomic structure of the human *ANKRD45* gene and sequences of two sgRNAs. Red arrows indicate the position and direction of the two sgRNAs. (**B**) Efficiency of the two sgRNAs analyzed by T7 endonuclease I digestion (T7EI). (**C**) Western blotting results showing the knockdown efficiency of two *ANKRD45*-targeting sequences. Both targets led to the reduction of ANKRD45 expression and increased the level of the cleaved PARP. (**D**–**F**) DIC images showing Hep3B cells transfected with control or *ANKRD45* targeting constructs containing different sgRNAs as indicated. Arrowheads represent the apoptotic cells. (**G**) Flow cytometry analysis of Annexin V-FITC and propidium iodide staining of Hep3B cells transfected with sgRNA or empty vectors. (**H**) Bar graph showing the percentages of Annexin V positive cells at 72 h and 96 h after transfection with control or sgRNAs as indicated. * *p* < 0.05, ** *p* < 0.01, Scale bar: 25 μm.

Considering that siRNA-mediated knockdown may cause off-target effects, we further designed two single-guide RNAs (sgRNAs) to transiently knockdown ANKRD45 expression using the CRISPR/Cas9 system (Figure 5A). The efficiency of these two sgRNAs was confirmed by T7EI assay (Figure 5B). Western blot with ANKRD45 antibody showed that the expression of ANKRD45 was successfully knocked down in both sgRNA transfected cells (Figure 5C). Again, we observed a large number of dead cells at 72 h after sgRNA transfection (Figure 5D–F). We stained the transfected cells with Annexin V, which further confirmed the increase of apoptotic cells (Figure 5G,H). Finally, the expression level of the cleaved form of PARP-1 was also upregulated when ANKRD45 was knocked down (Figure 5C).

Together, both the siRNA- and sgRNA-mediated knockdown results suggest that ANKRD45 plays an important role during cell division, and ANKRD45 deficiency can lead to cell proliferation defects and finally result in cell apoptosis, similar to results seen in zebrafish *ankrd45* mutants.

## 4. Discussion

Ankyrin repeat-containing proteins are one of the most common protein families present in eukaryotic cells and they play a role in various biological functions. Here, we reported the function of a novel ANK protein, ANKRD45, which contains two ankyrin repeats. The expression pattern of *ankrd45* in early zebrafish embryos was tissue specific with high enrichment in ciliated organs, suggesting that Ankrd45 possibly plays a role during cilia development. Indeed, ANKRD45 was also identified recently from proteomics analysis by comparison of ciliary proteins in different species [29]. Unexpectedly, homozygous *ankrd45* mutants were viable and showed no apparent defects. All the cilia detected were grossly normal in *ankrd45* mutants. Although we cannot rule out subtle defects, our results suggest that Ankrd45 is not essential for ciliogenesis in zebrafish. Interestingly, *ankrd45* mutants displayed abnormal reaction during stress response, such as Kras^G12V^-induced tumor formation. Overexpression of the oncogenic Kras^G12V^ could promote tumor formation when strongly expressed in the liver in wild-type zebrafish [22]. The induction of enlarged livers seems to be more prominent in *ankrd45* mutant larvae. However, our histological results suggest that the enlarged livers were indeed due to the presence of a large amount of cystic degeneration due to cell apoptosis. These data suggest that Ankrd45 participates in the regulation of Kras^G12V^-induced cell proliferation. Similarly, ANKRD45 is also important for cell proliferation in several cancer cells, including human hepatoma Hep3B cells, which are relatively similar to the Kras^G12V^-induced liver cells.

Although deficiency of Ankrd45 proteins resulted in cell division defects both in Kras^G12V^-induced liver cells and cultured cancer cells, we did not observe any cell proliferation defects in *ankrd45* mutants. One possible explanation is that the embryos are complex, multicellular organisms with elaborate gene regulation systems, which can trigger multiple signaling pathways to compensate for the loss of Ankrd45 in the mutant larvae [30]. Under stress conditions, such as rapid cell division in tumor cells, which might require high ANKRD45 levels, the gene regulation systems fail to fully compensate for the loss of ANKRD45, which eventually leads to cell apoptosis. The high requirement of ANKRD45 during cancer cell proliferation may be worthy of further attention to evaluate the potential target effects during therapeutic trials.

The dynamic subcellular localization of ANKRD45 during cell cycle progression is consistent with its role in cell proliferation. Time-lapse microscopy showed that cell division was arrested during the cell cycle, resulting in cell death at interphase and/or metaphase, which suggested that ANKRD45 is required for cell division both in early and late mitotic stages. Currently, the mechanism by which ANKRD45 regulates mitosis is still unclear. ANKRD45 was slightly enriched beneath the plasmid membrane during the cleavage furrow formation and later in the midbody ring (Figure 3A–D”). Such a distribution was similar to that of some actin binding proteins, such as ezrin/radixin/moesin (ERM) proteins, which are involved in the regulation of membrane morphology [28,31]. Future studies will be necessary to decipher the mechanisms by which ANKRD45 regulates mitosis.

## Supplementary Materials

The following are available online at https://www.mdpi.com/2073-4425/10/6/462/s1, Figure S1: Identification of *ankrd45* mutants, Figure S2: Liver-specific induction of Kras^G12V^ in *ankrd45* mutants, Figure S3: Localization of ANKRD45 in different cell lines, Movie S1: Time-lapse microscopy of HeLa cells treated with control siRNA, Movie S2: Time-lapse microscopy of HeLa cells treated with *ANKRD45* siRNA.
