# Supplementary material for: Ankrd45 Is a Novel Ankyrin Repeat Protein Required for Cell Proliferation
Source: Genes (Basel). 2019 Jun 16;10(6):462. doi: 10.3390/genes10060462 (PMC6628321; doi:10.3390/genes10060462)
Supplement: Supplementary file 1 [file genes-10-00462-s001.zip › Figure Suppl text-190527.pdf]

Figure S1. Identification of *ankrd45* mutants

(A) Sequences of partial *ankrd45* cDNA from wild type and mutant (*Δ5*) alleles. Red box indicates the 5bp sequence that was absent in the mutant allele. Bottom shows the predicted proteins encoded by mutant and wild type *ankrd45* alleles. (B) Sample electrophoresis images showing the genotype results of wild type, heterozygous and homozygous zebrafish larvae. (C-D) Whole in situ hybridization results of *ankrd45* expression in wild type (C) and *ankrd45* homozygous mutants (D). The number of embryos examined was shown in the bottom. Scale bar: 100 μm.

Figure S2. Liver-specific induction of *Kras*^{G12V} in *ankrd45* mutant

(A) Dotted graph showing the average size of liver in different groups as indicated. Ectopic expression of Ankrd45 inhibited the overgrowth of liver in the homozygous mutants (MT). The number of embryos examined was shown in the bottom. (B) Confocal images showing the apoptotic cells stained by TUNEL assay at 4 and 5 days after treatment. (C) Confocal images showing proliferating cells visualized by BrdU incorporation assay. (D) Statistical results of apoptotic cells at different time point after doxycycline treatment in the liver of control and *ankrd45* larvae. (E) Statistical results showing the number of BrdU positive cells at different time point after doxycycline treatment. Scale bars, 25 μm.

Figure S3. Localization of ANKRD45 in different cell lines

(A-D) Confocal images showing the localization of ANKRD45 in the midbody ring during cytokinesis of different cells as indicated. (E-F) Pre-incubation with synthesized ANKRD45 protein blocked the staining of ANKRD45 antibody in the midbody ring of U2OS cells. (G) Western blotting results showing the expression of ANKRD45 and cleaved PARP in control or four *ANKRD45* siRNAs treated HeLa Cells. (H) Bar graph showing the relative expression level of ANKRD45 in control or siRNA treated HeLa cells. Scale bar: 5 μm.

Movie1

Time-lapse microscopy of HeLa cells treated with control siRNA

Movie2

Time-lapse microscopy of HeLa cells treated with *ANKRD45* siRNA
