# Supplementary figures and images for: Ankrd45 Is a Novel Ankyrin Repeat Protein Required for Cell Proliferation
Source: Genes (Basel). 2019 Jun 16;10(6):462. doi: 10.3390/genes10060462 (PMC6628321; doi:10.3390/genes10060462)

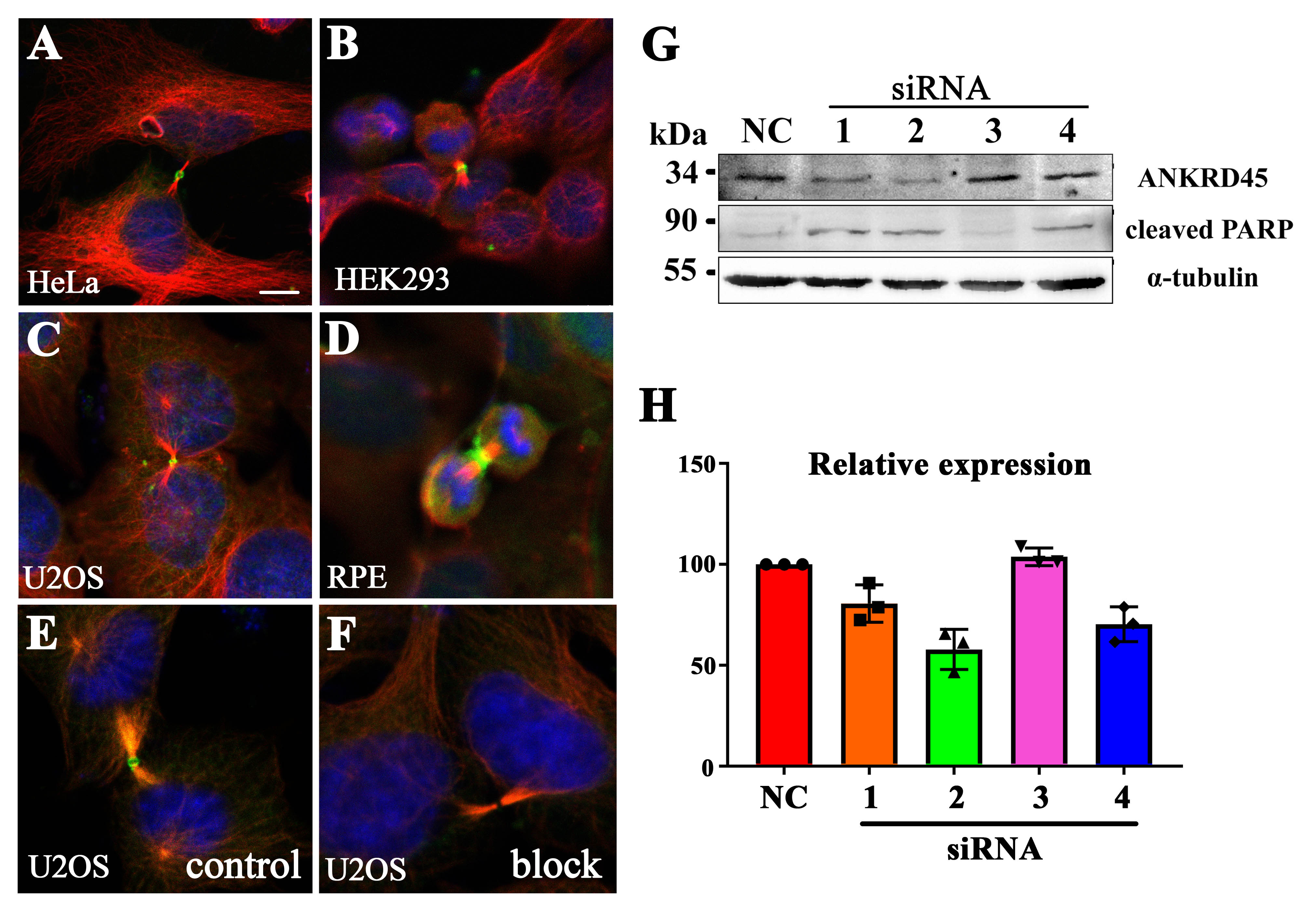

Supplement: Supplementary file 1 [file genes-10-00462-s001.zip › FigS3-190522.jpg]

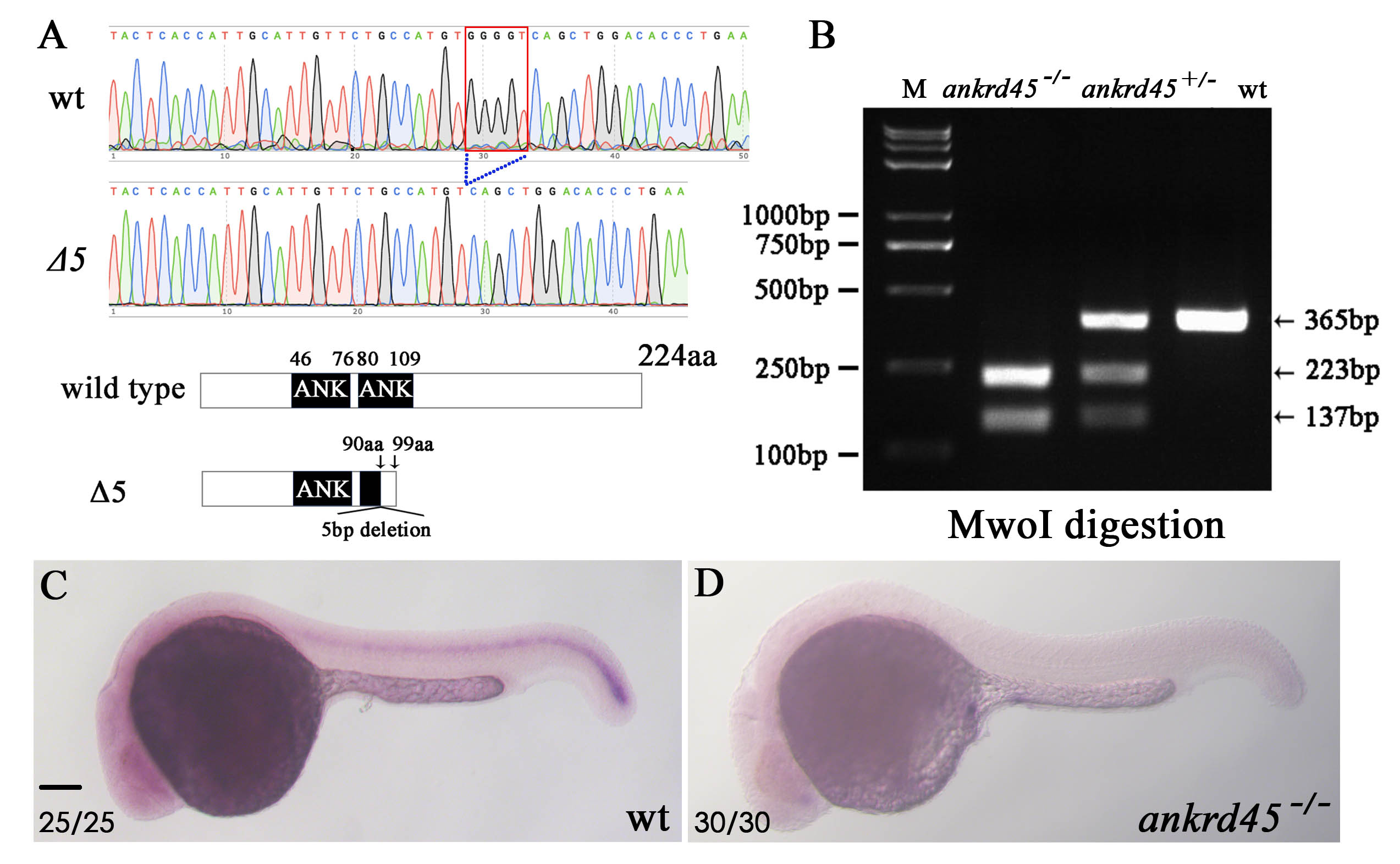

Supplement: Supplementary file 1 [file genes-10-00462-s001.zip › FigS1-190519.jpg]

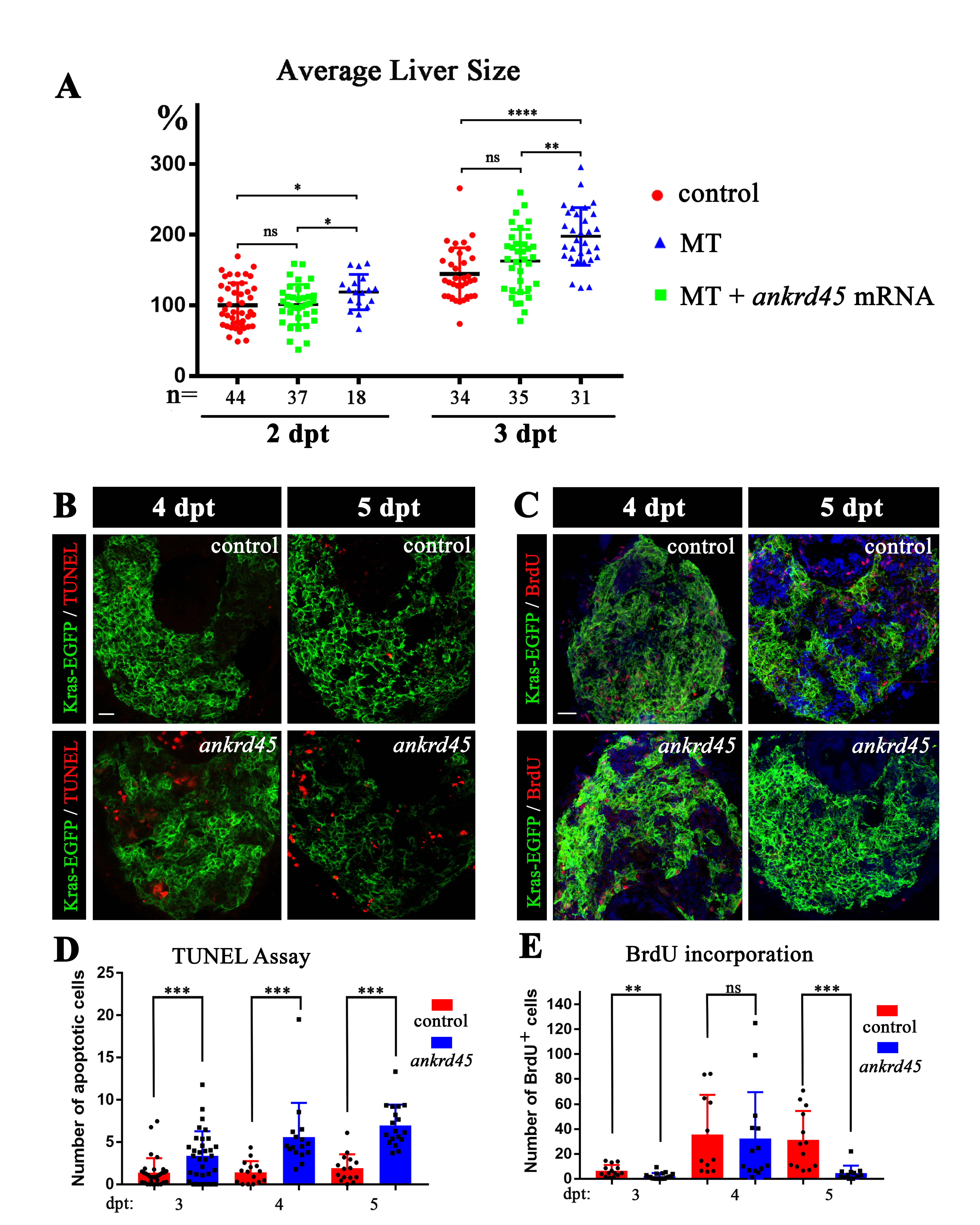

Supplement: Supplementary file 1 [file genes-10-00462-s001.zip › FigS2-190521.jpg]
